# Case Report: Successful Reperfusion of Pulmonary Thromboembolism Using tPA in a Cat

**DOI:** 10.3389/fvets.2022.851106

**Published:** 2022-04-20

**Authors:** Blake Sutton, Erin Long Mays, Chris McLaughlin

**Affiliations:** ^1^Veterinary Specialty Services, St. Louis, MO, United States; ^2^Department of Cardiology, College of Veterinary Medicine, North Carolina State University, Raleigh, NC, United States

**Keywords:** pulmonary thromboembolism, tPA, thrombolysis, fibrinolytic, echocardiography, feline

## Abstract

Pulmonary thromboembolism is a potentially life threatening condition that is uncommonly recognized in cats. Thrombolytic agents have been described as a treatment for this condition in human and canine patients, particularly in cases where hemodynamic instability is persistent despite supportive care. This report describes the clinical course, echocardiographic diagnosis, and successful thrombolysis of a cat with pulmonary thromboembolism. Despite confirmed reperfusion, the cat succumbed to thromboembolic disease highlighting the dearth of knowledge about optimal treatment of this disease process in small animals, particularly in cats.

## Background

Pulmonary thromboembolism (PTE) is an umbrella term that describes either the presence of a thrombus formed within a pulmonary arterial vessel (pulmonary thrombosis) or the mobilization of a thrombus (or fragment) from another site to the pulmonary vasculature (pulmonary embolism, or PE). Pulmonary thromboembolism in cats is a rare and sometimes fatal complication often associated with concurrent disease (1, 2). Prevalence is reported at 0.06% of cats seen over a 24 year period at a veterinary teaching hospital (1). This is likely an underestimate because the diagnosis may be infrequently considered and can be difficult to confirm. Pulmonary thromboembolism is thought to be the result of a combination of physiologic and hemostatic derangement represented by Virchow's Triad (hypercoagulability, endothelial injury, and blood stasis) which shifts the normal hemostatic balance toward a predisposition of clotting. Disease processes associated with PTE in cats include neoplasia, pancreatitis, anemia and cardiomyopathy (1, 2).

The severity of pulmonary and cardiac compromise that results from PTE can vary dramatically. Acute, massive PTE (defined as PTE causing hypotension and shock) is a life-threatening emergency, while chronic or submassive PTE may result in mild to moderate, non-specific clinical signs (3). The pulmonary vascular occlusion caused by massive PTE ultimately leads to myocardial dysfunction and right ventricular failure as the right ventricular myocardium is no longer able to overcome the acute rise in pulmonic outflow pressure.

Thrombolytic therapy has been used with variable success in small animals to treat severe thrombotic disease including acute feline aortic thromboembolism (2), canine aortic thromboembolism (3), catheter-associated thrombosis (4), and obstructive intravesicular blood clot formation (5). Systemic thrombolysis has been reported in clinical cases of canine PTE using an older thrombolytic agent (streptokinase) (6) but only experimentally using the more modern thrombolytic drug, recombinant tissue plasminogen activator (tPA) (7). To the authors' knowledge, there are no reports describing the use of systemic thrombolysis for the treatment of feline PTE. This report describes the clinical course, echocardiographic diagnosis, and successful thrombolysis of a cat with PTE causing severe hemodynamic and respiratory instability. In this cat, thrombolytic therapy resulted in significant, yet temporary, clinical improvement in the post-thrombolysis period.

## Case Presentation

A 9 year old spayed female domestic longhair cat was presented to a referral hospital following acute onset tachypnea and open-mouth breathing. The cat was originally presented to a local emergency center. There, she was sedated with butorphanol (0.2 mg/kg IM) to facilitate thoracic radiographs. Thoracic radiographs were performed and a definitive diagnosis was not apparent. The patient was treated with furosemide (2 mg/kg IV) and dexamethasone-SP (0.09 mg/kg IV). The patient was further sedated with an unrecorded dose of propofol to perform venipuncture. An automated CBC revealed a mild thrombocytopenia (110 K/uL, RI 151–600 K/uL), hyperglycemia (343 mg/dL, RI 71–159 mg/dL) and mild hypokalemia (3.3 mmol/L, RI 3.5–5.8 mmol/L). On recovery from sedation, the cat was significantly hypothermic at 33.1°C (91.6°F) and was subsequently referred to a specialty hospital.

On presentation to the specialty hospital emergency center, the cat was sedate, hypothermic (34.3°C), bradycardic (120 bpm), and tachypneic (86 rpm) with pale pink mucous membranes. Doppler blood pressure was measured at 105 mmHg. Electrocardiography revealed a sinus bradycardia with intermittent ventricular ectopy of left bundle branch block morphology. Physical exam revealed mildly increased lung sounds in all fields with no cardiac murmur. At presentation, blood glucose was 399 mg/dL, PCV was 35% and total solids was 7.6 g/dL. A peripheral venous blood gas revealed a pH of 7.25, pCO_2_ 46 mmHg, HCO_3_ 20 mEq/L, lactate 5.4 mmol/L, and BE −6.9. Thoracic radiographs were interpreted and revealed a left extrapleural mass, diffuse bronchial and unstructured interstitial pattern, cardiomegaly, and enlarged caudal lobar arteries relative to veins. Left sided congestive heart failure was suspected and an emergency echocardiogram was ordered. Furosemide (2 mg/kg IV) was administered and the patient was placed in an oxygen cage.

An echocardiogram revealed moderate pulmonary hypertension based on characteristic changes including a mild right atrial dilation, mild right ventricular eccentric hypertrophy and an estimated pulmonary arterial pressure measured at 55 mmHg, which may have been an underestimate in the presence of right ventricular systolic dysfunction. The main pulmonary artery appeared normal but the right branch of the pulmonary artery was moderately dilated (with some impairment to clear visualization noted). The left ventricular heart volume was underloaded. During the echocardiogram, ECG revealed sinus bradycardia with no ventricular ectopy. The cause of dyspnea and pulmonary hypertension was not identified and no cardiac medications were prescribed. Additional imaging was recommended following hemodynamic and respiratory stabilization. The cat was admitted to the intensive care unit with persistent hypothermia, tachypnea and bradycardia. In the coming hours, more ventricular arrhythmias were noted and systolic blood pressure decreased to 85 mmHg. A continuous infusion of norepinephrine titrated to 0.3 mcg/kg/min resulted in no improvement in blood pressure. Due to persistent bradycardia, atropine (0.01 mg/kg IV) was administered with no improvement in heart rate or blood pressure. The norepinephrine infusion was subsequently discontinued and an epinephrine infusion was initiated at 0.05 mcg/kg/min. Systolic blood pressure rose to 109 mmHg but dyspnea worsened and respiratory rates ranged from 100 to 120 breaths per minute with open-mouth breathing, despite oxygen supplementation. Venous blood gas revealed worsening respiratory acidosis (pH 7.2, pCO_2_ 62 mmHg, and HCO_3_ 25 mmol/L) and due to impending respiratory failure and hemodynamic instability, mechanical ventilation was recommended. Ketamine (5 mg/kg IV), midazolam (0.5 mg/kg IV), and fentanyl (5 mcg/kg IV) were administered to facilitate intubation. Atracurium (0.2 mg/kg IV) was administered shortly after induction due to severe patient-ventilator dyssynchrony. Thereafter, fentanyl, midazolam and atracurium were administered as continuous rate infusion. The patient was ventilated using synchronized intermittent mandatory ventilation with pressure control-pressure support setting. Peak pressure limit was set at 20 cmH_2_O, positive end expiratory pressure at 3 cmH_2_O, and frequency of 20 breaths per minute with an initial FiO_2_ of 100%. Pulse oximetry readings remained low (89% progressing to 74%) despite FiO_2_ ranging 70–100%.

**Figure 1:**
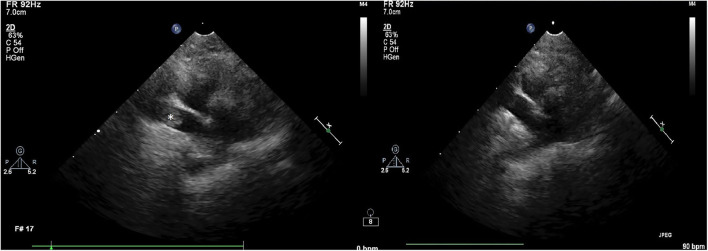
Two-dimensional (2D) right parasternal echocardiographic image on first examination (left) showing the presence of a thrombus (noted by the *) and repeat image (right) showing successful thrombolysis.

Due to persistent hypoxemia despite high FiO_2_ positive pressure ventilation, the ICU clinician performed bedside lung and focused cardiac ultrasound and detected a large, occlusive thrombus in the right pulmonary artery. This finding was confirmed on subsequent echocardiogram performed by a board certified cardiologist (see Figure 1). Because of refractory hypoxemia and hemodynamic instability requiring epinephrine infusion, systemic thrombolysis was recommended. Prior to thrombolysis, platelet count was assessed by automated CBC and confirmed manually at 117 × 10^3^/μL (RR 198–434). A kaolin-activated thromboelastogram (TEG) revealed a prolonged R time (6.5 min, RI 2–5 min) and a mildly decreased alpha angle (55.6 deg, RI 58–75 deg). These findings signal mildly decreased coagulation factor, fibrinogen, and (to a lesser extent) platelet activity (8). Although decreased enzyme and platelet production or function was considered as an explanation for this tracing, it was thought more likely to be a result of enzyme and platelet consumption in this patient with confirmed thrombosis. After the risk of drug-induced hemorrhage was conveyed to the family, thrombolysis was elected. Local vs. systemic thrombolysis was considered in this case. However, placement of a pulmonary arterial catheter was not possible within an acceptable time frame. Because of the cat's instability, the clinicians prioritized immediate, systemic thrombolysis over a delayed catheter-directed method, despite the potential benefit of reduced hemorrhage risk associated with the latter. The cat was blood typed (type AB) and blood product inventory was confirmed to be sufficient in case of hemorrhage.

Cathflo^®^ Activase^®^ (alteplase), a recombinant tissue plasminogen activator, was administered at 1 mg/kg IV via central venous catheter over 1 h with the first 10% of the infusion administered as an IV bolus over 10 min as per institutional protocol for acute feline aortic thromboembolism. Upon completion of the infusion, the patient was evaluated for any evidence of external or cavitary hemorrhage and none was detected. A baseline whole blood aPTT was performed and was prolonged (212 s, RI 60–115 s). Despite this, in order to prevent reocclusion or other thrombotic events, a continuous IV infusion of unfractionated heparin was instituted at a fixed dosage of 600 U/kg/day. No loading dose was administered. Pulse oximetry readings rose to 91% during infusion and 94% by completion of tPA infusion (FiO_2_ 60%). FiO_2_ was further titrated down to 50% and SpO_2_ was stable in the mid 90s. The epinephrine infusion was titrated down from 0.15 mcg/kg/min to 0.05 mcg/kg/min and subsequently discontinued. Blood pressure was maintained >100 mmHg systolic throughout the taper and remained stable following discontinuation. Follow-up echocardiography revealed reperfusion of the previously occluded right pulmonary artery (Figure 1) and alleviation of pulmonary hypertension with resolution of the previously present thrombus.

Whole blood aPTT was performed for the purposes of heparin monitoring and revealed prolongation outside of the institutional reference range. For this reason, the unfractionated heparin CRI was discontinued. Evaluation for hemorrhage was performed via point-of-care ultrasound and external examination but none was detected. Seven hours post-thrombolysis, the patient became acutely bradycardic and went into cardiopulmonary arrest. Because hemorrhage was not detected on current or recent exams and clinical decline was peracute, the clinician on duty suspected recurrent PTE and alteplase was re-dosed as a bolus during resuscitation. Resuscitation attempts were unsuccessful. Necropsy was performed and revealed severe pulmonary arterial thrombosis in the right and left caudal lobar arteries (right worse than left). No hemorrhage was detected. There was no mass noted in the thoracic cavity as initially suspected on thoracic radiographs. Systemic disease factors contributing to the development of PTE were not detected on necropsy and the cause remained unidentified.

## Discussion

This report documents that systemically administered tPA is efficacious in achieving thrombolysis for feline pulmonary thromboembolism at the dose described. There are several other unique features about this case that contribute to current literature including the means by which the thrombus was diagnosed and the lack of sustained antithrombotic therapy which may have contributed to reocclusion.

Pulmonary thromboembolism in small animals is rare and is often difficult to definitively diagnose. Particularly in cases where shock and severe respiratory distress are present, options for diagnostic testing that the patient can withstand are limited (9). Diagnosis of PTE is further challenged by the non-specific clinical signs and variable severity associated with the condition. Patients often present with pulmonary and cardiovascular sequelae that are reflective both of the degree of pulmonary vascular occlusion and the individual's ability to compensate for such changes (9). No laboratory test can confirm PTE. Thoracic radiographs may reveal pulmonary vascular changes, other non-specific changes, or, in up to 25% of dogs, no change at all (1, 6, 9). Computed tomographic pulmonary angiography (CTPA) is the preferred imaging modality for diagnosing pulmonary embolism in people (10) with growing support for use in small animals (11). However, sedation or general anesthesia is often necessary in veterinary patients to optimize CT imaging results. Hypotension is considered a common complication during anesthesia (12), and many drugs used solely or in combination for sedation and anesthesia can have deleterious effects on heart rate, blood pressure, and ventilation among other parameters (13, 14). This may pose unacceptable risk for hemodynamically unstable patients when alternative methods of diagnosis are available.

Echocardiography offers a safe, non-invasive alternative to CTPA that may reveal cardiac changes associated with a PTE, such as evidence of pulmonary hypertension, right ventricular dilation, interventricular septal flattening and, occasionally, direct visualization of a thrombus within a pulmonary artery (9). However, with the exception of direct visualization of the thrombus, the findings remain non-specific. Given the speed and safety of echocardiography, this diagnostic approach is increasingly favored in the evaluation of human patients suspected of having massive pulmonary embolism and, where available, could lead to the diagnosis in small animals without need for sedation, anesthesia, or iodinated contrast administration (15). In this case, the diagnosis of PTE was initially made via bedside focused cardiac ultrasound and confirmed using echocardiography. However, the clot was not identified on initial echocardiogram, raising the question of this modality's sensitivity for PTE diagnosis. Factors such as patient movement (as was reported here) and operator experience may affect sensitivity. Timing of the thrombotic event may also affect echocardiographic diagnosis as an acute thrombus may be hypoechoic initially, followed by a transition to hyperechogenicity over time (16, 17). In this case, it is also possible that the clot was not present at the time of the first echocardiogram, although an alternative cause of pulmonary hypertension could not be identified.

Veterinary guidelines describing the optimal management of clinically important PTE have not been published. Therefore, treatment strategies for the management of PTE in small animals are largely extrapolated from human guidelines. In unstable patients, initial management is focused on optimizing hemodynamics and providing respiratory support. This is achieved through careful management of obstructive shock, early pharmacologic intervention for hypotension and right ventricular failure, the provision of supplemental oxygen, and in cases of respiratory failure, mechanical ventilation (10). The benefit of mechanical ventilation must be carefully weighed against the potential for negative hemodynamic consequences of anesthetic induction, as mentioned previously, and positive pressure ventilation (PPV) (14). Specifically, high intrathoracic pressures during PPV can reduce venous return and worsen cardiac output. And, of particular concern in cases of PTE, lung over-distention from mechanically delivered tidal volumes can further increase pulmonary vascular resistance and worsen right ventricular failure (18).

Apart from management of hemodynamic instability and respiratory distress, prevention of on-going clot formation must be prioritized as thrombus propagation can result in progressive pulmonary vascular occlusion. In people highly suspected to have a pulmonary embolism, this is accomplished by early treatment with parenteral anticoagulants such as unfractionated or low molecular weight heparin, even diagnosis is not yet confirmed (10). There is insufficient evidence to determine superiority of one anticoagulant over the other for PTE treatment in small animals, but continuous IV unfractionated heparin is often preferred in people in whom primary reperfusion therapy (systemic thrombolysis) is imminent because it's effect can be more rapidly eliminated once discontinued (10).

Reperfusion therapy can be achieved via systemic thrombolysis using a fibrinolytic agent, commonly recombinant tissue plasminogen activator (tPA). Fibrinolytic agents act directly on plasminogen, facilitating its conversion to the active enzyme plasmin which is capable of degrading fibrin. In addition to acute massive pulmonary embolism, systemic thrombolysis is also used in people to resolve other life-threatening thromboses such as acute myocardial ischemia or acute ischemic stroke (19). Local thrombolysis using catheter-directed techniques can also be used for some of these indications as well as in non-life threatening conditions such as extremity venous or arterial thrombosis or catheter-associated thrombosis (19).

In people with pulmonary embolism, systemic thrombolysis is typically reserved for cases in which the patient is hemodynamically unstable or experiencing right ventricular dysfunction (10, 19). Thrombolytic treatment, ideally in the first 48 h, can result in faster improvement to hemodynamic variables compared to unfractionated heparin alone in people (20). A meta-analysis of human thrombolysis trials revealed a reduction in mortality and PE recurrence in patients treated with systemic thrombolysis (21). However, it is important to note that any benefit of systemic thrombolysis comes at the cost of increased risk for hemorrhage. There are many absolute and relative contraindications to thrombolysis in people including active bleeding, history of stroke, recent (within three 3 months) trauma, surgery or head injury, CNS neoplasm, non-compressible vessel puncture, active peptic ulcer, among others (10, 19). In small animals, such contraindications have not been defined.

Although this report documents successful reperfusion of pulmonary thromboembolism using tPA in a cat, this reperfusion was not sustained. The lack of consistent antithrombotic therapy following thrombolysis may have been a contributing factor. Anticoagulation following thrombolysis is a key component in the maintenance of reperfusion and is standard of care in humans treated with thrombolysis for unstable pulmonary embolism (19). Though there are no comparable studies involving venous thrombosis, numerous canine coronary artery reperfusion studies have documented that anticoagulation has a favorable effect not only on time to thrombolysis but also in delaying or preventing reocclusion after successful thrombolysis (22–25). The prolongation of aPTT out of range was the obvious reason for discontinuation of heparin therapy as one would expect that therapeutic anticoagulation had been achieved and risk of bleeding was high. However, in hindsight, the cat's subsequent death due to reocclusion raises the question of possible other causes for such a dramatic aPTT prolongation including lab error or Factor XII deficiency. Even prior to anticoagulation, the cat demonstrated a significant prolongation in aPTT and delayed clot formation on kaolin activated TEG. While a consumptive coagulopathy could explain these derangements, it is possible that Factor XII deficiency could have caused similar results (26).

## Conclusion

To the authors' knowledge, this report documents the first use of systemic thrombolysis in a cat as a treatment for PTE. The subsequent improvement in hemodynamic variables and documented reperfusion on repeated echocardiogram suggests that this therapeutic approach could be beneficial in the management of cats with severe, hemodynamically unstable PTE, as recommended in human guidelines. However, the failure of sustained reperfusion highlights the importance of future research to define optimal post-thrombolysis anticoagulation protocols. There are limitations to this study, the greatest of which is that only a single case is reported. Therefore, one cannot conclude that all cats will respond similarly to the described therapy or diagnostic approach. Ideally, higher level of evidence, non-descriptive studies should to determine if thrombolytic therapy results in improved patient-centered outcomes in acute pulmonary thromboembolism.

## Data Availability Statement

The original contributions presented in the study are included in the article/supplementary material, further inquiries can be directed to the corresponding author/s.

## Ethics Statement

Written informed consent was obtained from the owner of the cat for the publication of this case report.

## Author Contributions

BS and EL contributed to writing the manuscript and literature review. CM provided images. All authors reviewed and edited the manuscript.

## Conflict of Interest

The authors declare that the research was conducted in the absence of any commercial or financial relationships that could be construed as a potential conflict of interest.

## Publisher's Note

All claims expressed in this article are solely those of the authors and do not necessarily represent those of their affiliated organizations, or those of the publisher, the editors and the reviewers. Any product that may be evaluated in this article, or claim that may be made by its manufacturer, is not guaranteed or endorsed by the publisher.
